# Impact of Sensitization and Inflammation on the Interaction of Mast Cells With the Intestinal Epithelium in Rats

**DOI:** 10.3389/fphys.2019.00329

**Published:** 2019-03-26

**Authors:** Jasmin Becker, Daniela Ott, Martin Diener

**Affiliations:** Institute for Veterinary Physiology and Biochemistry, Justus-Liebig-University Giessen, Giessen, Germany

**Keywords:** claudins, colitis, inflammation, intestinal secretion, mast cells

## Abstract

The density of intestinal mast cells has been reported to increase during inflammatory bowel disease (IBD). As mast cell mediators are known to increase the permeability of epithelial tight junctions, we hypothesized that antigen responses in sensitized animals might be enhanced under inflammatory conditions. This would contribute to a vicious circle by further enhancing the entry of luminal antigens into the colonic wall and thereby continuing the inadequate immune response during IBD. Therefore, one group of rats was sensitized against ovalbumin. In a second group of animals additionally a colitis was induced by rectal administration of 2,4,6-trinitrobenzenesulfonic acid (TNBS) dissolved in ethanol. Specimens from distal colon and jejunum (as intestinal segment located distantly from the inflamed area) were mounted in Ussing chambers to measure tissue conductance, short-circuit current (I_sc_) induced by antigen exposure and paracellular permeability (fluorescein flux). This was paralleled by determination of mast cell markers and tight junction proteins with immunofluorescence and qPCR. In contrast to the initial hypothesis, antigen-induced I_sc_ was not upregulated, but tended to be downregulated in the tissues from the colitis animals, both in colon and in jejunum. Only in the jejunum mast cell degranulation evoked an increase in fluorescein flux. Mast cell density was not altered significantly in the colon of the colitis animals. In the jejunum, sensitization induced a strong increase in mast cell density, which was unaffected by additional induction of colitis. Expression of sealing tight junction components claudin-3 and -4 were increased on the protein level in the sensitized animals in comparison to non-sensitized animals. Additional induction of colitis evoked a downregulation of claudin-3 in both intestinal segments and an upregulation of claudin-4 in the jejunum. Consequently, these data indicate segment differences in mast cell – epithelium interaction, but no enhancement of ion secretion in the TNBS/ethanol model of acute colitis after prior sensitization.

## Introduction

Mast cells are bone marrow-derived cells at the interface between the innate and the adaptive immune system. The FcεRI on the surface of mast cells is able to bind immunoglobuline E (IgE), which can recognize and bind antigens. The consequence is the secretion of mast cell mediators inducing further immune responses with the final aim to remove the antigen. About 2–3% of all cells in the lamina propria of the intestinal mucosa are mast cells; this amount can increase up to 10-fold during intestinal disorders (Bischoff, 2009). Intestinal mast cells of rodents can be subdivided into mucosal mast cells and connective tissue mast cells due to their location in the intestinal wall (van Nassauw et al., 2007) and exhibit a high heterogeneity (see e.g., Gallwitz et al., 2007). They contain different mediators either preformed (and stored in mast cell granules) or synthesized *de novo* on demand. Histamine, serotonin, tryptase, serine proteases, proteoglycans, and tumor necrosis factor-alpha (TNFα) belong to the preformed mediators, whereas prostaglandins, leukotrienes, platelet activating factor, and several cytokines will be synthesized *de novo* (De Winter et al., 2012).

Various stimuli, especially crosslinking between antigens, IgE and the FcεRI, lead to a degranulation of mast cells (van Nassauw et al., 2007). Centrally involved in this response is an increase in the cytosolic Ca^2+^ concentration. Mast cell mediators act in a paracrine manner on neighboring cells, e.g., submucosal neurons (Schemann and Camilleri, 2013; Buhner et al., 2017). In the intestine of rats these neurons can be activated, among others, by histamine via H_1_ receptors and to a smaller extend via H_2_ receptors (Bell et al., 2015). Activation of secretomotor neurons lead to a secretion of Cl^-^ followed by water into the gut lumen. A further action site is the epithelium, where mast cell mediators, such as proteases, increase the paracellular permeability (Scudamore et al., 1995; Fernández-Blanco et al., 2015), e.g., by affecting tight junction proteins like claudin-2 (Amasheh et al., 2002). Consequently, mast cells are a central cell type involved in food allergy (Brandt et al., 2003).

However, also during inflammatory bowel diseases (IBD) and irritable bowel syndrome (IBS) a role of mast cells has been shown (De Winter et al., 2012; Wouters et al., 2016). For IBDs like Crohn’s disease or ulcerative colitis different environmental, genetic and immunological factors contribute to their pathogenesis. The clinical symptoms vary between secretory diarrhea, abdominal pain and weight loss and the therapy options are not always effective. Although the pathogenesis is not fully understood, it is generally assumed that an impaired intestinal epithelial barrier is one of the first steps in the pathogenesis of IBD (Wittkopf et al., 2014). This is accompanied by a higher density of mast cells in the intestinal wall (Rijnierse et al., 2007). Hence, different effects on neuronal and epithelial cells due to the altered mast cell mediator release or receptor composition are seen in the inflamed intestinal tissue.

Chronic intestinal inflammation leads to a dysregulation of the barrier function and increases the permeability of the epithelium. During this process, among others, the proinflammatory cytokine TNFα is involved, which is stored in mucosal mast cells (Keita and Söderholm, 2010). To prevent the uncontrolled passage of ions and water, the apicolateral membrane of the enterocytes contains tight junction proteins sealing the paracellular space (Barmeyer et al., 2017). One prominent member of the tight junction protein family are claudins, which can be functionally subdivided into sealing, pore-forming or ambiguous proteins (Markov et al., 2017). In rodents, claudins 1–19 are differently expressed in the epithelium of small and large intestine depending on the segment or villus-crypt axis (Günzel and Yu, 2013). During IBD, their expression is either increased or decreased depending on the claudin type (Markov et al., 2010; Garcia-Hernandez et al., 2017).

Mast cell density has been reported to be increased during IBD (Rijnierse et al., 2007). Based on the central role of mast cells in food allergy, we hypothesized that allergen responses *in vitro* should be enhanced by prior induction of a colitis. Such an effect might contribute as a kind of vicious circle to the pathogenesis of IBD, as mast cell mediators reduce the epithelial barrier function, which would further enhance the contact of immune cells with luminal antigens. Consequently, in the present study we compared ion secretion evoked by antigen exposure *in vitro* in Ussing chambers in rats sensitized against ovalbumin (OVA-sensitized group) and rats, which had in addition been treated with TNBS/ethanol in order to induce an acute colitis (OVA+colitis group). In this model of colitis, ethanol serves as barrier disrupting agent allowing the hapten TNBS to bind to subepithelial proteins.

Although it has been reported that topical administration of ethanol alone does not induce a macroscopically recognizable colitis (for review see Antoniou et al., 2016), we use the term TNBS/ethanol model, as probably also the contact with microbiota after barrier disruption might contribute to the colitis in this experimental setup. However, the principal aim of this study was not to find out the contribution of ethanol to the overall response in this IBD model, but to compare mast cell – epithelium interactions in small and large intestine of rats with and without colitis. Thus, we did not compare the response to antigen exposure in sensitized animals with and without TNBS/ethanol with an additional group of animals treated with ethanol alone.

## Materials and Methods

### Animals

Male and female Wistar rats with an age of 6–8 weeks were used. The animals were bred and housed at the institute for Veterinary Physiology and Biochemistry of the Justus Liebig University Giessen at an ambient temperature of 22.5°C and air humidity of 50–55% on a 12 h : 12 h light-dark cycle with free access to water and food during the experiment. The animals were anaesthetized with isoflurane and killed by exsanguination. Experiments were approved by Regierungspräsidium Giessen, Giessen, Germany (administrative number GI 18/2 Nr. 3/2016) and performed according to the German and European animal welfare law.

### Solutions

For the Ussing chamber experiments a bathing solution was used containing 107 mM NaCl, 4.5 mM KCl, 25 mM NaHCO_3_, 1.8 mM Na_2_HPO_4_, 0.2 mM NaH_2_PO_4_, 1.25 mM CaCl_2_, 1 mM MgSO_4_ and 12.2 mM glucose. The solution was gassed with carbogen (5% CO_2_ and 95% O_2_, v/v) and kept at a temperature of 37°C. The immunofluorescence experiments and the fixation were carried out with phosphate buffer containing 80 mM Na_2_HPO_4_ and 20 mM NaH_2_PO_4_. For the toluidine blue staining, 1 g toluidine blue was dissolved in 100 ml aqua dest. The pH of all solutions was adjusted to 7.4 with NaOH (1 M) or HCl (1 M).

### Sensitization

**FIGURE 1:**
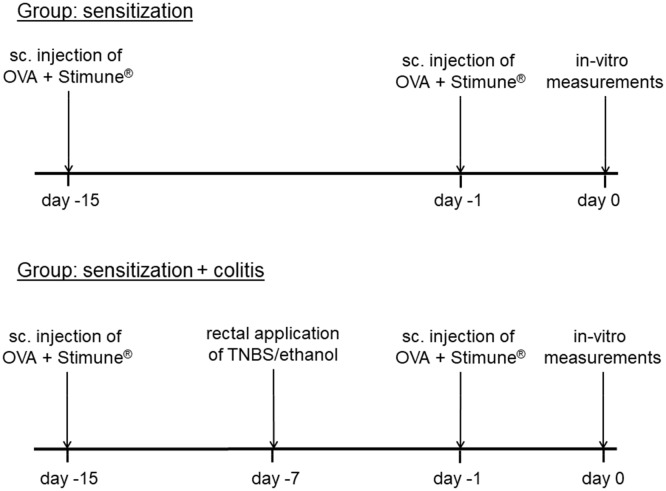
Schematic time schedule of the colitis/sensitization experiment. Sc, subcutaneous, TNBS, 2,4,6-trinitrobenzenesulfonic acid dissolved in ethanol. All animals were sensitized by subcutaneous injection of ovalbumin (OVA) combined with Stimune^®^ on day -15 and -1. In the second group (lower tracing), a mild colitis was induced additionally with TNBS/ethanol at day -7 before the animals were euthanized.

Rats were sensitized against ovalbumin (OVA) by repeated subcutaneous injection of a solution containing 10 μg ovalbumin (grade V; Sigma, Taufkirchen, Germany) dissolved in 50 μl sterile 0.9% (w/v) NaCl and 60 μl of the adjuvant Stimune^®^ (Prionics, Zurich, Switzerland). Stimune^®^ (specol) is a selected water-in-oil emulsion (for composition see Bokhout et al., 1981), which has less side effects compared to e.g., Freund’s adjuvant (Leenaars et al., 1994). In our hands, also sensitization rates against ovalbumin were increased from 81 to 88% (Hug et al., 1996; Amstutz and Diener, 1997) to 100% (Bell et al., 2015), when replacing Freund’s adjuvant against Stimune^®^. The subcutaneous injections were applied on days -15 and -1 before starting with *in vitro* measurements (Figure 1). All animals were successfully sensitized as indicated by the consistent increase of the short-circuit current (I_sc_) evoked by antigen exposure in Ussing chamber experiments (see Results), which is not observed in tissues from non-sensitized animals (Hug et al., 1996). For the morphological and molecular biological measurements, in addition samples from untreated, i.e., non-sensitized animals were taken as control group.

### Induction of Colitis With TNBS/Ethanol

In one group of the sensitized animals (OVA+colitis), a colitis was induced by the rectal application of 10 mg 2,4,6-trinitrobenzenesulfonic acid (TNBS) dissolved in 250 μl 50% (v/v) ethanol 8 days (day -7) before euthanasia of the animals (Figure 1). Under isoflurane anesthesia (2%, v/v) the TNBS-solution was administered rectally into the colonic lumen at a distance of 8 cm from the anus by a catheter with a diameter of 1 mm. To ensure that the solution remained in the colon, the animals were kept in a slight head-down position during and after the procedure for a few minutes. The rats were returned to their cages, where a heat source was offered until they woke up. All animals received flupirtine, a centrally acting, non-opioid analgesic via drinking water (100 mg⋅l^-1^) daily from day -7 until the end of the experiment, which does not interfere with the induction of colitis by TNBS/ethanol (Steidle et al., 2013). One week after TNBS/ethanol treatment, all rats developed a mild colitis as described in detail previously (Steidle et al., 2013). The health status of all rats was checked daily. Animals treated with TNBS/ethanol showed a transient weight loss. The normal increase in weight, which amounted to 4.3 ± 0.4 g⋅d^-1^ (*n* = 16) in the OVA-sensitized group, was reversed into a transient weight loss, which maximally amounted to -3.3 ± 2 g⋅d^-1^ (*n* = 12) in the OVA+colitis animals and recovered within a few days. During the week after the TNBS/ethanol administration, all rats of the OVA+colitis group exhibited a higher defecation frequency. After euthanasia and opening the abdomen, each animal showed a dilated colon and softer feces compared to the animals which had only been sensitized against ovalbumin without additional induction of a colitis. We did not perform a histological disease index because all specimens taken from the animals were used for functional Ussing chamber experiments, immunofluorescent staining or qPCR analysis. However, the model used here has been characterized in detail (including histological scoring and myeloperoxidase assay) in a previous study (Steidle et al., 2013).

### Tissue Preparation

One segment of jejunum (taken at a distance of 10 cm oral from the ileocaecal valve) and one segment of distal colon were used from each animal. Due to their small thickness, the jejunal segments were mounted in Ussing chambers as whole-mount, i.e., unstripped preparations with all layers of the intact intestinal wall (see below). For the colonic preparations with their thicker intestinal wall, muscle-stripped mucosa-submucosa preparations were used to reduce the diffusion barrier. Therefore, the distal colon was put over a plastic rod, a circular incision was made near the distal end of the colon and the serosa and the muscularis propria were removed carefully by hand in proximal direction. The tissue was opened lengthwise at the mesenteric side and was cut in the required size for each experiment.

For immunofluorescent staining of mast cells pulmonary tissue was chosen as reference tissue. Therefore, the part near to the diaphragm of the caudal pulmonary lobes was removed, rinsed, fixed and stained as described below. Samples for qPCR were stored in RNAlater (Macherey-Nagel, Düren, Germany).

### Ussing Chamber Experiments and Fluorescein Flux Measurements

The tissue was fixed in a modified Ussing chamber, which was filled with 3.5 ml bathing solution on each side and tempered at 37°C. For electrophysiological measurements the tissue was short-circuited by a computer-controlled voltage-clamp (Mussler, Aachen, Germany). The tissue conductance (G_t_; in mS⋅cm^-2^) was measured every minute. The short-circuit current (I_sc_; in μEq⋅h^-1^⋅cm^-2^ with 1 μEq⋅h^-1^⋅cm^-2^ = 26.9 μA⋅cm^-2^) expresses the flux of monovalent ions per time and area. Secretion of anions (or electrogenic absorption of cations) is reflected by a positive I_sc_.

Experiments were started after a stabilization phase of 30 min. During the recording of I_sc_ and G_t_, also the flux of fluorescein was measured in parallel. Therefore, fluorescein (10^-4^ M at mucosal side), which passes the epithelium via the paracellular way, was administered after the stabilization phase. Samples (volume: 70 μl) were taken six times in 30 min intervals (t0; t30; t60; t90; t120; t150) from the serosal side. After t60, ovalbumin (100 μg⋅ml^-1^ at mucosal and serosal side) was administered and at the end of each experiment carbachol (5⋅10^-5^ M at serosal side) was added as viability control. All aliquots from the serosal side were replaced by fluorescein-free buffer solution and appropriate correction for this replacement solution was performed.

After centrifugation of the samples (11,000 min^-1^, 10 min), their extinction was measured with Nanodrop One© (Thermo Fisher Scientific, Lafayette, CO, United States) at a wavelength of 488 nm. The flux of fluorescein (expressed in nmol⋅h^-1^⋅cm^-2^) was calculated from the difference in fluorescein concentration between two adjacent time points. The fluxes of the two control periods (from t0 to t30 and from t30 to t60) were averaged for each tissue and served as reference point to measure the changes in paracellular permeability induced by antigen exposure.

### Immunofluorescence

The tissue was fixed in phosphate buffer containing 4% (w/v) paraformaldehyde and stored at 4°C overnight. Then the tissue was embedded in gelatine (100 g⋅l^-1^) and cryofixed in N_2_-cooled isopentane. Four microgram thick sections were mounted on microscope slides (Superfrost^®^ Plus, Thermo Fisher Scientific) alternately either for immunofluorescent or for toluidine blue staining, so that serial sections were obtained. After rehydration, the sections were incubated for 2 h in a blocking solution containing phosphate buffer with 0.2% (v/v) triton-X-100, 3% (w/v) bovine serum albumin (BSA), and 10% (v/v) donkey or goat serum depending on the host, in which the secondary antibody was produced. Each primary antibody was dissolved in phosphate buffer containing 0.1% (v/v) Triton-X-100, 1% (w/v) BSA, 0.5% (w/v) milk powder, and 1% (v/v) donkey or goat serum. The sections were incubated with the primary antibody overnight at 4°C (for sources and final dilutions, see Table 1). After repeated washing steps they were loaded with the secondary antibody for 1 h at room temperature and were washed again. The primary antibodies against c-kit and claudin-1, -3, -4, and -8 (Markov et al., 2010) were combined with the secondary antibody donkey anti-rabbit IgG (Cy3-coupled). For nuclear staining the sections were incubated with 3⋅10^-7^ M 4,6-diamidio-2-phenylindoldilactate (DAPI) for 5 min and embedded with Hydromount^®^ (Biozym, Oldendorf, Germany) in case of the mast cell staining. During the claudin staining, RotiFluo with DAPI (Roth, Karlsruhe, Germany) was used for the nuclear staining and embedding. Pictures were taken with the fluorescence microscope Nikon 80i (Nikon, Düsseldorf, Germany). All claudin antibodies, which gave positive signals in immunohistochemical staining (Figure 5, 6), were validated by Western blot. For claudin-3 and claudin-4 positive bands were observed at ∼23 kDa, for claudin-8 at ∼27 kDa. For the c-kit antibody used in immunohistochemichal staining (Figure 4), no signal was observed in Western blots, which is probably due to the low number of mast cells in the intestinal wall in relation to other cell types from which the isolated proteins used for the blot originate. For all immunohistochemichal series of experiments, specificity of the secondary antibody was tested by incubation of the tissue without the respective primary antibody (see Figure 5, 6).

**Table 1:** Primary and secondary antibodies used for immunofluorescent staining.

Target	Host	Supplier	Final dilution
**Primary antibodies**		
C-kit	Rabbit	Santa Cruz (sc-168)	1:250
Claudin-1	Rabbit	Invitrogen (51–9000)	1:500
Claudin-3	Rabbit	Invitrogen (34–1700)	1:1000
Claudin-4	Rabbit	Invitrogen (36–4800)	1:500
Claudin-8	Rabbit	Invitrogen (40–0700Z)	1:1000 (colon) 1:2000 (jejunum)
Histamine	Rabbit	Sigma (H7403)	Up to 1:250
Mast cell tryptase (AA1)	Mouse	Abcam (ab2378)	Up to 1:250
Mast cell tryptase (AA1)	Mouse	Santa Cruz (sc-59587)	Up to 1:100
**Secondary antibodies**		
Mouse IgG (Alexa 488-coupled)	Donkey	Invitrogen (A121202)	Up to 1:250
Mouse IgG (Cy3-coupled)	Goat	Dianova (115-165-003)	Up to 1:250
Rabbit IgG (Cy3-coupled)	Donkey	Dianova (711-165-152)	1:1000


For mast cell counting, three randomly selected slides from three different animals per group (untreated control group, OVA-sensitized and OVA+colitis) were analyzed in a blinded fashion. Mast cells were counted independently from their localization, i.e., mucosal and submucosal mast cells were included. The length and the average height of the sections was measured using NIS Elements 2.30 software (Nikon) and the mast cells were normalized to 1 mm^2^ of the tissue.

For the comparison of claudin expression levels (Figure 5, 6), sections from each group were stained in parallel under the same conditions. Randomly taken pictures from both sites were analyzed by a researcher in a blinded fashion. All photographs for a single claudin were taken with the same setting (same exposure time and amplification) of the camera. Areas were randomly selected in the surface epithelium for quantitative analysis of the Cy3 signal using ImageJ (version 1.50i^1^) and the color intensity was measured in arbitrary units.

### Toluidine Blue Staining of Mast Cells

For the detection of mast cells, sections of distal colon and jejunum were stained in a 1% (w/v) aqueous toluidine blue solution. The 4 μm thick sections were rehydrated in phosphate buffer and incubated for 1 h in toluidine blue. Then the slices were washed in 70% (v/v) ethanol, 100% (v/v) isopropanol and RotiHistol^®^ (Roth), in each for a few seconds, and finally embedded with Hydromount^®^. Pictures were taken with the light microscope Nikon 80i.

### Quantitative Real-Time Polymerase Chain Reaction (qPCR)

For qPCR experiments jejunum and mucosa-submucosa preparations of distal colon were transferred in RNAlater, homogenized with a mixer mill and the total RNA was extracted by using the RNA Plus kit (Macherey-Nagel) including columns to remove genomic DNA. The amount of each RNA sample (ng⋅μl^-1^) and their purity (OD260/280) was determined with the Nanodrop One© (Thermo Fisher Scientific). Before starting qPCR, the integrity of the RNA samples was determined by gel electrophoresis. An equal amount of RNA (250 ng⋅μl^-1^) was transcribed to cDNA with High-Capacity RNA-to-cDNA Kit (Thermo Fisher Scientific). For each experimental condition and tissue the qPCR experiments were performed with TaqMan^®^ Gene Expression Master Mix (Thermo Fisher Scientific) by using cDNA diluted 1:10. NormFinder (MOMA, Denmark; Andersen et al., 2004) was used to define two reference genes (out of 16 tested), which showed the most stable expression. For distal colon NormFinder defined Gapdh and B2m as the two most stable expressed genes in all three groups (control animals, sensitization, and sensitization combined with colitis) and Ppia and Rplp2 for jejunum (Table 2). The relative expression of three different mast cell proteases was measured (rMCPt-2 = chymase, rMCPt-6 and rMCPt-7 = both tryptase subunits) according to their distribution in mucosa (mast cells containing chymase; rMcpt-2) and in submucosa (mast cells containing tryptase; rMcpt-6 and rMcpt-7). Furthermore, the relative expression of two claudins (claudin-3, claudin-4) was measured with qPCR (StepOne Plus, Applied Biosystems). All primer were obtained from Thermo Fisher Scientific (for further information see Table 2). qPCR was performed in two independent series of experiments; for each primer three technical replicates and three (sensitization group) or six (control and colitis group) biological replicates were used. The individual efficiency for each gene was calculated with LinRegPCR^2^. All calculated efficiencies were between 1.8 and 2.0. A mean efficiency per amplicon group was determined and outliers (more than 5% of the mean) were excluded. The baseline corrected Cq values were used for further evaluation and statistics with REST^®^ (2009) as described by Pfaffl et al. (2002) for group-wise comparison of qPCR data.

**Table 2:** Overview of primers used for qPCR.

Target gene	Assay-ID	Gene accession number
rMCPt-2	Rn00756479_g1	NM_172044.1
Tpsb2 ( = rMCPt-6)	Rn00569857_g1	NM_019180.2
Tpsab1 ( = rMCPt-7)	Rn00570928_m1	NM_019322.2
Cldn3	Rn01499274_s1	NM_031700.2
Cldn4	Rn01196224_s1	NM_001012022.1
Gapdh	Rn99999916_s1	NM_017008.4
B2m	Rn00560865_m1	NM_012512.2
Ppia	Rn00690933_m1	NM_017101.1
Rplp2	Rn01479927_g1	NM_001030021.1


*rMCPt, rat mast cell protease (rMCPt-2, chymase); Tpsb2, tryptase beta-2; Tpsab1, tryptase alpha/beta 1; Cldn, claudin; Gapdh, glyceraldehyde-3-phosphate dehydrogenase; B2m, beta-2-microglobulin; Ppia, peptidylprolyl isomerase A (cyclophilin A); Rplp2, ribosomal protein, large P2.*

### Drugs

Carbachol, fluorescein and ovalbumin (grade V) were dissolved in aqueous stock solutions. If not indicated differently, drugs were from Sigma, Taufkirchen, Germany.

### Statistics

Results are given as mean ± standard error of the mean (SEM) with the number of investigated tissues or cells (n). To compare two groups, paired or unpaired *t*-test or a Mann-Whitney *U*-test was performed. In order to find out, which test method had to be used, *F*-test was performed. For the comparison of more than two groups, an analysis of variance (ANOVA) was applied. This was followed by Fishers Least Significant Difference (LSD) *post hoc* test. The decay of the I_sc_ induced by carbachol was fitted to a mono- or biexponential function using GraphPad Prism 5 (GraphPad Software, La Jolla, CA, United States). Quality of regressions was determined by the squared non-linear regression coefficient (*r*^2^). The qPCR results were statistically analyzed with REST Software (Pair Wise Fixed Reallocation Randomization Test©). *P* < 0.05 was considered to be statistically significant.

## Results

### Sensitization and Inflammation Affect Anion Secretion and Epithelial Permeability

**FIGURE 2:**
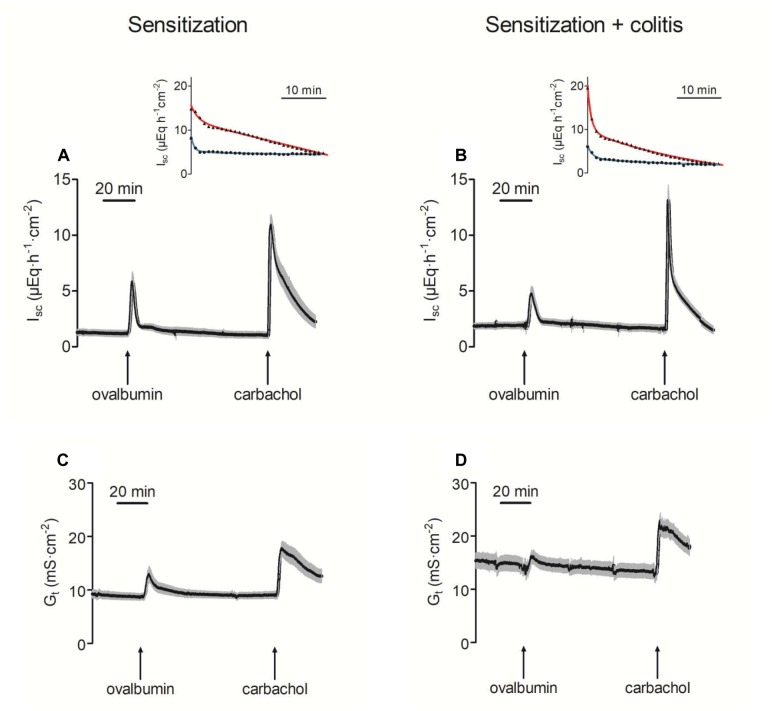
Increase of short-circuit current (I_sc_; **A,B**) and tissue conductance (G_t_; **C,D**) in distal colon after administration of ovalbumin (100 μg⋅ml^-1^ at mucosal and serosal side) in animals sensitized against ovalbumin without **(A,C)** or with additional induction of a colitis with TNBS/ethanol **(B,D)**. The final administration of carbachol (5⋅10^-5^ M at serosal side) served as viability control. Values are means (black area) ± SEM (gray area), *n* = 8–11. The insets show a representative original tracing of the decay of the carbachol-induced I_sc_ in each experimental group (red line = distal colon; blue line = jejunum). Black points in the insets are typical I_sc_ traces from individual tissue (reduced to one data point every minute for graphical clarity). The colored lines show the fit to an exponential function (for time constants and statistics, see Table 4).

In Ussing chamber experiments, stimulation of mast cells was achieved by administration of ovalbumin (100 μg⋅ml^-1^ at the mucosal and the serosal side) to tissues from animals sensitized against this protein. In all tissues tested, ovalbumin evoked a prompt increase in I_sc_ (Figure 2 and Table 3), which amounted to 5.71 ± 1.02 μEq⋅h^-1^⋅cm^-2^ (P < 0.05 versus baseline, *n* = 9) in the distal colon and 3.35 ± 0.41 μEq⋅h^-1^⋅cm^-2^ (*P* < 0.05 versus baseline, *n* = 8) in the jejunum of sensitized animals (OVA-sensitized). This was concomitant with a significant increase in tissue conductance (G_t_) in both intestinal segments (Figure 2 and Table 3). Colonic tissues from rats, which in addition had been treated rectally with TNBS/ethanol to induce colitis (OVA+colitis), showed a slightly elevated basal I_sc_ (not statistically significant) and the G_t_ was significantly elevated (14.13 ± 1.25 mS⋅cm^-2^, *n* = 11, compared to 8.69 ± 0.63 mS⋅cm^-2^, *n* = 9 without colitis, *P* < 0.05).

**Table 3:** Ussing chamber data of distal colon and jejunum.

		OVA-sensitization	OVA+colitis
	
Distal colon	I_sc_ (μEq⋅^-1^⋅cm^-2^)		
	Baseline	1.20 ± 0.29	1.92 ± 0.32
	ΔI_sc_ ovalbumin	5.71 ± 1.02^∗^	3.44 ± 0.65^∗^
	ΔI_sc_ carbachol	11.11 ± 0.92^∗^	13.24 ± 1.20^∗^

	**G_t_ (mS⋅cm^-2^)**		

	Baseline	8.69 ± 0.63	14.13 ± 1.25^#^
	ΔG_t_ ovalbumin	4.10 ± 0.98^∗^	3.23 ± 0.49^∗^
	ΔG_t_ carbachol	9.26 ± 0.85^∗^	10.94 ± 1.28^∗^

**Jejunum**	**I_sc_ (μEq⋅h^-1^⋅cm^-2^)**		

	Baseline	1.64 ± 0.45	1.92 ± 0.51
	ΔI_sc_ ovalbumin	3.35 ± 0.41^∗^	2.28 ± 0.39^∗^
	ΔI_sc_ carbachol	1.51 ± 0.26^∗^	2.09 ± 0.31^∗^

	**G_t_ (mS⋅cm^-2^)**		

	Baseline	32.20 ± 2.17	38.57 ± 3.45
	ΔG_t_ ovalbumin	4.37 ± 0.91^∗^	6.26 ± 1.78^∗^
	ΔG_t_ carbachol	8.39 ± 1.48^∗^	11.02 ± 3.32^∗^


*Short-circuit current (I_*sc*_) and tissue conductance (G_*t*_) of distal colon (upper part) and jejunum (lower part) in OVA-sensitized animals (middle column) and OVA+colitis animals (right column). Given are the baseline just prior administration of ovalbumin (i.e., after 90 min) and the maximal increase in I_*sc*_ (ΔI_*sc*_) or G_*t*_ (ΔG_*t*_) induced by ovalbumin (100 μg⋅ml*^-^*^*1*^ at mucosal and serosal side) or carbachol (5⋅10*^-^*^*5*^ M at serosal side). ΔI_*sc*_ and ΔG_*t*_ are given as maximal change of the parameter above baseline just prior administration of the respective agonist. Data expressed as mean ± SEM, n = 8–11. ^∗^P < 0.05 versus baseline (paired t-test), ^#^P < 0.05 versus same parameter in the sensitization group (unpaired t-test).*

In both intestinal segments from the OVA+colitis animals, the response to ovalbumin was not enhanced. There was a trend for a reduction by 30–40%, although these differences did not reach statistical significance (Table 3). In other words, there was no upregulation of antigen-induced secretion in the colitis group (but rather an apparent downregulation).

The Ca^2+^-dependent secretagogue carbachol (5⋅10^-5^ M at the serosal side) evoked a significant increase in I_sc_ and G_t_. Neither in the colon nor in the jejunum the amplitude of this response was altered in the OVA+colitis group (Figure 2 and Table 3). The current induced by carbachol shows a transient time course and falls due to an active downregulation with a biexponential time course in rat colon (Strabel and Diener, 1995). Close inspection of the decaying phase revealed a change in the time course of the carbachol-evoked current, which obviously fell quicker to basal values in the OVA+colitis group. Fitting this decay to a biexponential function (inset in Figure 2A,B) with a fast and a slow time constant (τ) revealed that τ_fast_ was strongly reduced from 6.32 ± 2.86 min (*n* = 9) in the OVA-sensitized group to 0.93 ± 0.08 min (*n* = 11) in the OVA+colitis group (Table 4). In contrast to the colon, in the jejunum carbachol evoked an increase in I_sc_ which decayed quickly to baseline values within a few minutes. This decay could be fitted with a monoexponential function and the time constant was slightly prolonged in OVA+colitis animals (Table 4).

**Table 4:** Kinetics of the carbachol response.

Conditions		τ_fast_ (min)	τ_slow_ (min)
Distal colon	Sensitization	6.32 ± 2.86	43.27 ± 14.04
	Sensitization + colitis	0.93 ± 0.08^#^	35.69 ± 12.34
Jejunum	Sensitization	0.82 ± 0.09	–
	Sensitization + colitis	1.30 ± 0.21^#^	–


*The decay of the I_*sc*_ induced by carbachol (5⋅10^-*5*^ M at serosal side) was fitted to a biexponential function with a slow and fast time constant (τ) in the distal colon and a monoexponential function in the jejunum (see Figure 2, inset). r^*2*^ for all fits ranged from 0.70 to 0.99. Data expressed as mean ± SEM, n = 8–11. ^#^P < 0.05 versus same parameter in the sensitization group (unpaired t-test or U-test).*

**FIGURE 3:**
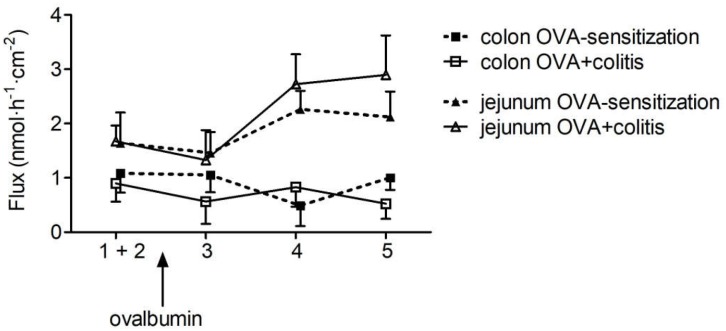
Flux of fluorescein from the mucosal to the serosal side in five sequential 30 min periods in rat jejunum (triangles) and colon (squares) of OVA-sensitized animals with (open symbols) or without (filled symbols) prior induction of a colitis. After determination of basal fluorescein flux in two control periods, which were pooled for statistical analysis (period 1 + 2), ovalbumin (100 μg⋅ml^-1^) was administered at the mucosal and serosal side and the fluorescein flux was measured in three further 30 min periods (period 3–5). For graphical clarity, data were laterally shifted and SEM is only given as +SEM or -SEM to avoid overlay of the symbols. Data expressed as mean (symbols) + or - SEM (vertical lines), *n* = 9–12.

The increase in G_t_ evoked by ovalbumin indicates a higher permeability of the epithelium for ions. In order to find out whether this was correlated with an increased permeability of the paracellular pathway for small, uncharged molecules, the flux of the low molecular weight marker fluorescein was measured. Administration of ovalbumin (100 μg⋅ml^-1^ at the mucosal and the serosal side) evoked an increase in the fluorescein flux in the jejunal segments, which was not different between both experimental groups. In contrast, in colon the fluorescein flux remained constant in both groups after administration of ovalbumin (Figure 3).

### Segment-Dependent Differences on Intestinal Mast Cells

**FIGURE 4:**
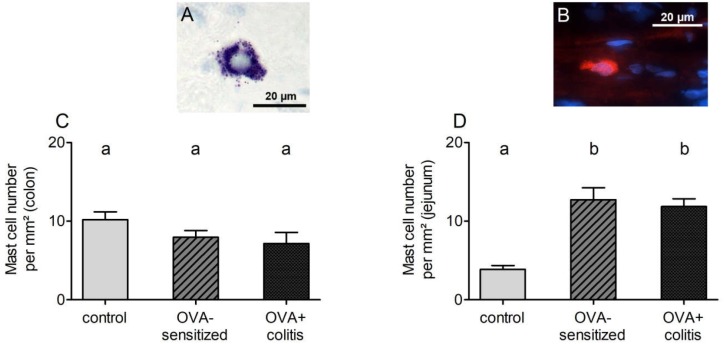
Exemplary images of submucosal mast cells stained with toluidine blue (**A**, from a control animal) or c-kit (**B**, from a sensitized animal). Mast cell density (c-kit positive cells per mm^2^) quantified in distal colon **(C)** or jejunum **(D)**. Data expressed as mean (bars) + SEM (lines), *n* = 9 sections analyzed with a total number of 152–530 mast cells. Statistically homogeneous groups are labeled with the same letter (a, b), thus all groups which do not share the same letter are significantly different from each other. *P* < 0.05 (ANOVA followed by LSD *post hoc* test).

In order to find out whether sensitization or sensitization in combination with the TNBS/ethanol colitis model alters mast cell density in comparison to untreated control animals, different staining techniques were tried for the identification of mast cells within the wall of the small or large intestine. Staining of mast cell granules with toluidine blue stained only a few submucosal mast cells in the control group (Figure 4A). However, in the two experimental groups (OVA-sensitized and OVA+colitis group) no reliable staining was obtained (data not shown), which might suggest a stronger degree of mast cell degranulation. Therefore, several immunofluorescent staining with antibodies against typical mast cell markers were tested to find out, which of them resulted in a consistent labeling of rat intestinal mast cells. Rat pulmonary tissue served as positive control for the protocols used. Two different antibodies against mast cell tryptase (for primary antibodies used, see Table 1) and an antibody against histamine stained mast cells in the pulmonary tissue, but not in the intestinal tissue (data not shown). A reliable staining of intestinal rat mast cells, however, was possible with an antibody against the surface marker c-kit (CD117, stem-cell factor receptor) (Figure 4B). Consequently, this marker was selected for further analysis.

C-kit positive cells (outside the tunica muscularis to avoid mismatch with c-kit positive interstitial cells of Cajal) were analyzed morphometrically. Mast cell density in the colon amounted to 10.2 ± 1.0 per mm^2^ in the control group and decreased by 25% in the two experimental groups (Figure 4C). However, this decrease did not reach statistical significance. A different pattern was observed in jejunum. Here, mast cell density was more than three times higher in the OVA-sensitized group compared to the control group (Figure 4D). Additional induction of colitis did not alter significantly jejunal mast cell density in comparison to the OVA-sensitized group.

### Altered Epithelial Barrier Function

The Ussing chamber experiments revealed a significant increase in G_t_ in the OVA+colitis group compared to the OVA-sensitized animals and a trend for an increased G_t_ in the jejunum (Table 3). In order to find out, whether this might correlate with changes in the expression of different barrier-forming claudins, immunofluorescent staining against claudin-1, -3, -4, and -8 were performed. We selected those four claudins, which have been shown in rat to represent the member of the claudin family with a strong segment-specific expression along the length of the intestine with its known gradient in transepithelial resistance (low in small intestine, high in colon; Markov et al., 2010).

**FIGURE 5:**
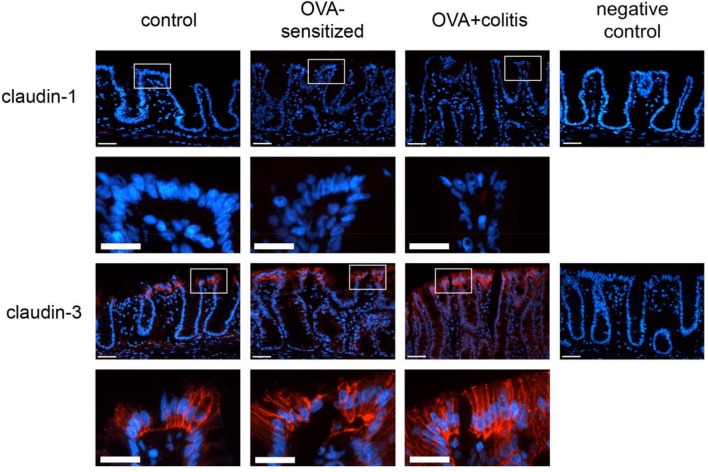
Expression of claudin-1 (upper two rows) and claudin-3 (lower two rows) in rat colon under control conditions (1st column), in OVA-sensitized animals (2nd column), and in OVA+colitis animals (3rd column). The 4th column shows negative controls where the primary antibody against the respective claudin was omitted. Row 2 and 4 show enlargements of the pictures presented in the respective upper rows (with a rectangle depicting the localization of the magnifications). Thin calibration bar (1st and 3rd row): 50 μm; thick calibration bar (2nd and 4th row): 25 μm. Representative staining of three independent experiments for each claudin type.

**FIGURE 6:**
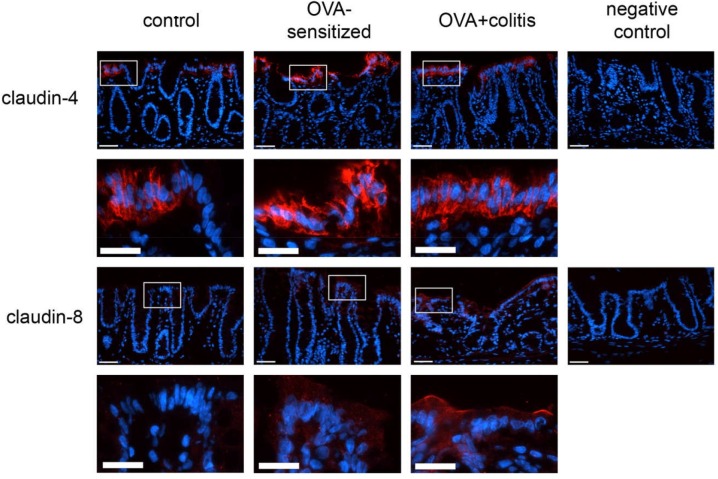
Expression of claudin-4 (upper two rows) and claudin-8 (lower two rows) in rat colon under control conditions (1st column), in OVA-sensitized animals (2nd column), and in OVA+colitis animals (3rd column). The 4th column shows negative controls where the primary antibody against the respective claudin was omitted. Row 2 and 4 show enlargements of the pictures presented in the respective upper rows (with a rectangle depicting the localization of the magnifications). Thin calibration bar (1st and 3rd row): 50 μm; thick calibration bar (2nd and 4th row): 25 μm. Representative staining of three independent experiments for each claudin type.

**FIGURE 7:**
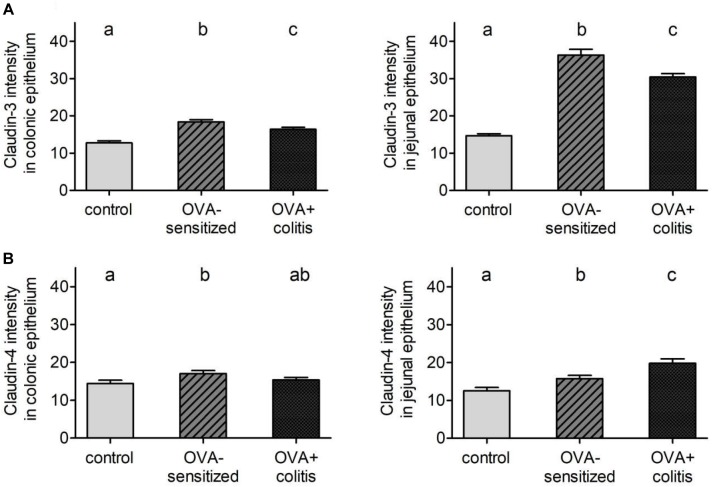
Morphometric analysis of claudin-3 **(A)** and -4 **(B)** levels under control conditions (gray bars), in OVA-sensitized animals (crossed bars), and in OVA+colitis (black bars) in distal colon (left) and jejunum (right). Data expressed as mean (bars) + SEM (lines), *n* = 163–401 cells in each group were counted. Statistically homogeneous groups are labeled with the same letter (a, b, c), thus all groups which do not share the same letter are significantly (*P* < 0.05) different from each other (ANOVA followed by LSD *post hoc* test).

In our hands, we did not obtain any positive result in immunofluorescent staining with the antibody used against claudin-1 (Figure 5), whereas for the other three claudins immunoreactivity in the epithelium was observed. Immunoreactivity was absent in the negative controls, where the primary antibody was omitted (right columns in Figure 5, 6). In the colon as well as in the jejunum, the claudin-3 immunofluorescence (Figure 5) increased significantly in OVA-sensitized animals (*P* < 0.05; Figure 7). In the specimens from OVA+colitis animals the immunofluorescence was decreased compared to the OVA-sensitized group, but was still significantly higher than in segments from control animals (Table 5).

A different pattern was observed for claudin-4 (Figure 6). Sensitization increased the claudin-4 immunofluorescence intensity significantly (*P* < 0.05) in comparison to the control group in both intestinal segments. However, in the colonic samples from OVA+colitis animals its level remained constant in the colon but further increased in the jejunum (Figure 7 and Table 5).

**Table 5:** Quantification of claudin-3 and claudin-4 expression intensity.

	Control	OVA-sensitization	OVA+colitis
**Claudin-3 signal intensity**			
Distal colon	12.73 ± 0.58^a^	18.41 ± 0.59^b^	16.36 ± 0.57^c^
Jejunum	14.69 ± 0.53^a^	36.30 ± 1.57^b^	28.84 ± 0.85^c^
**Claudin-4 signal intensity**			
Distal colon	14.41 ± 0,86^a^	17.03 ± 0.79^b^	15.40 ± 0.65^ab^
Jejunum	12.53 ± 0.86^a^	15.74 ± 0.85^b^	19.89 ± 1.18^c^


*Increased immunofluorescence intensity of claudin-3 and -4 in the experimental groups compared to control. Claudin intensity was quantified by morphometric measurement of gray levels (Figure 4). Values are means ± SEM. A total number (n) of 163–401 cells from slides from three different animals were analyzed. Statistically homogeneous groups are labeled with the same letter (a, b, c), thus all groups which do not share the same letter are significantly different from each other. P < 0.05 (ANOVA with LSD post hoc test).*

Claudin-8 distribution along the epithelium was discontinuous. It was only expressed in apical membrane of the inflamed intestinal epithelium (Figure 6). Due to the discontinuous expression, no morphometric analysis was performed.

### Gene Expression Levels of Mast Cell Proteases and Sealing Claudins

**FIGURE 8:**
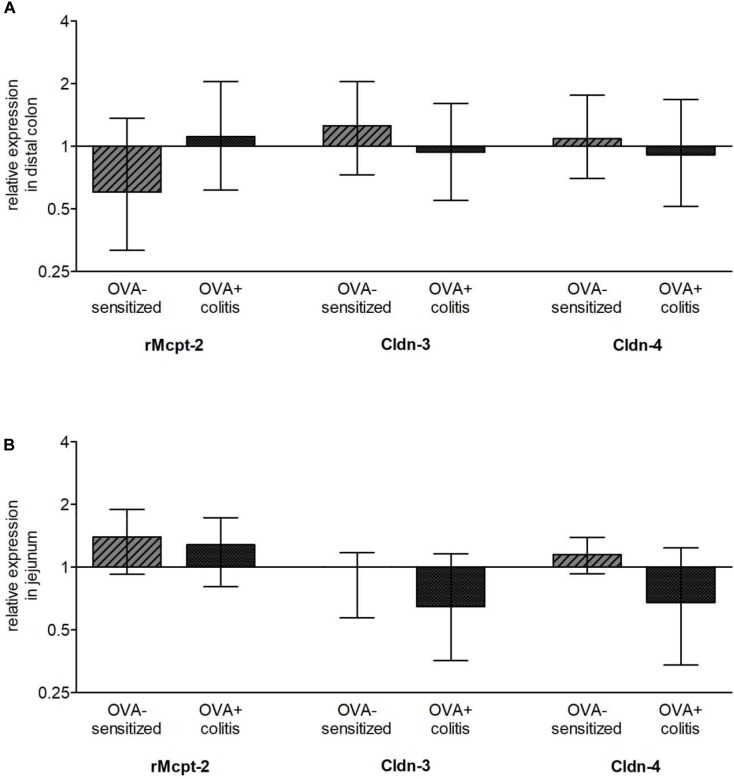
Relative expression of chymase (rMcpt-2), claudin-3 (Cldn-3), and claudin-4 (Cldn-4) in comparison to control animals in distal colon **(A)** and jejunum **(B)**. Up- (ratio > 1) and downregulation (ratio < 1) are shown on a log2 scale. As stable expressed reference genes (defined by NormFinder) Gapdh and B2m for the distal colon and Ppia and Rplp2 for the jejunum were used. Data expressed as median (bars) ± SEM (lines), *n* = 3–6. None of these changes reached statistical significance when tested with REST^®^ (pair wise fixed reallocation randomization test©).

After determination of mast cells and claudins on the protein level, the gene expression of three different mast cell proteases and two claudins was measured with qPCR. The amplification of rMcpt-6 and rMcpt-7 started very late (more than 35 cycles) and did not reach a plateau in any of the samples tested so that they were excluded from further analysis. A reproducible amplification was observed for rMcpt-2 and was quantified in relation to its expression under control conditions in the colon or the jejunum, respectively. Sensitization tended to differently affect rMcpt-2 expression in the two intestinal segments: it decreased numerically in the colon (Figure 8A), but increased numerically in the jejunum (Figure 8B), however, these changes were not statistically significant. Also the levels of claudin-3 and -4 expressions on the mRNA level did not differ significantly in the two experimental groups, neither in the colon nor in the jejunum (Figure 8).

## Discussion

*In vitro* antigen exposure in tissues from sensitized animals evokes a strong Cl^-^ secretion induced by the release of mast cell mediators such as histamine, eicosanoids, proteases and others. These mediators can stimulate epithelial receptors and receptors on secretomotor submucosal neurons, which release prosecretory neurotransmitters such as acetylcholine (Wang et al., 1991; Javed et al., 1992). As mast cell density has been reported to be increased during inflammatory conditions (see e.g., Rijnierse et al., 2007), we hypothesized that the secretory response induced by mast cell degranulation might be enhanced after additional induction of a colitis. However, the present results demonstrate a contrary effect: the ovalbumin induced anion secretion (I_sc_) *in vitro* tended to be reduced after additional inflammation of the colon (mimicking for example colitis ulcerosa). Interestingly, this was observed not only for the colon, where a chemically induced colitis had been induced by topical application of TNBS/ethanol, but also in the small intestine, i.e., distant from the inflamed area. Interestingly, changes oral from the inflamed colonic area have already been observed in guinea pig ileum, where e.g., excitatory synaptic transmission in neurons expressing vasoactive intestinal peptide (VIP) was reduced, whereas the electrophysiological properties of the epithelium seemed to be unaltered (Hons et al., 2009).

Carbachol, a stable acetylcholine derivative, evoked a strong increase in I_sc_ both in segments from OVA-sensitized as well as from OVA+colitis animals. Hence, the reduced amplitude of the antigen-induced I_sc_ cannot be attributed to the known down-regulation of secretory epithelial transport mechanisms during inflammatory conditions (Martínez-Augustin et al., 2009). However, close inspection of the time course of the carbachol-induced I_sc_ reveals differences in the ability of the colonic epithelium to maintain secretion over a longer period of time. Carbachol induces anion secretion via stimulation of epithelial muscarinic M1 and M3 receptors coupled to an increase in the cytosolic Ca^2+^ concentration. The consequence is the activation of Ca^2+^-dependent K^+^ channels, which hyperpolarizes the membrane and thereby induces Cl^-^ secretion via an increased driving force for Cl^-^ exit via apical Cl^-^ channels (Böhme et al., 1991). In the colon, this I_sc_ is composed of two components, a fast and a slow decaying response (Strabel and Diener, 1995). This might represent different sources for Ca^2+^ mobilization, i.e., initial release from intracellular stores followed by a sustained influx via cation channels in the plasma membrane (Frings et al., 1999). The decay is dramatically accelerated during colitis as shown by the strong reduction of the time constant for the fast decaying phase (τ_fast_) by about 85%. If this is caused by a depletion of intracellular Ca^2+^ stores under inflammatory conditions, which represent the source of cytosolic Ca^2+^ during the early phase of the carbachol response (Lindqvist et al., 1998), remains to be determined. In the jejunum, carbachol-induced Cl^-^ secretion has a much shorter time course and decays with a monoexponential time constant of only about 1 min in the sensitization group, which was not further reduced, when colitis was induced additionally.

Likewise, baseline electrical properties of the epithelium are altered in OVA+colitis animals. As one should expect, there was a trend for a higher baseline I_sc_ (indicating a higher spontaneous anion secretion) in the colon of tissues from OVA+colitis animals. In addition, there was a significant increase in G_t_ by 60% indicating a higher permeability of the epithelium for ions, e.g., via the paracellular pathway. Interestingly, also the jejunum, which was not in contact with the topically applied TNBS/ethanol, showed a trend for a higher basal I_sc_ and a higher G_t_. This might suggest alterations in epithelial properties also in uninflamed areas, e.g., caused by circulating cytokines in the blood known to be elevated in patients with IBD, which can affect epithelial barrier properties (Hering and Schulzke, 2009).

An attractive hypothesis for the assumed role of mast cell mediators in IBDs is the idea that these mediators will increase the permeability of the tight junctions. Thereby, it allows a further influx of antigens from the intestinal lumen thus establishing a vicious circle contributing to an inadequate immune response during IBD. However, in a previous study (Bell et al., 2015), we could not detect an increase in the flux of fluorescein, a marker substance, that is only transported via the paracellular pathway after antigen contact in colonic segments from OVA-sensitized animals. Thus, the question was addressed in the present study, whether under inflammatory conditions, e.g., after the assumed changes in mast cell number and/or after preconditioning of the epithelium with proinflammatory cytokines, mast cell mediators might open tight junctions and thereby enhance fluorescein flux. This was, however, not the case in distal colon: antigen exposure let the fluorescein flux unaffected both in animals with or without colitis. This was different in the small intestine: ovalbumin increased the fluorescein permeability in the jejunum both in tissues from OVA-sensitized animals as well as OVA+colitis animals. These results might be caused by differences in the composition (see e.g., Markov et al., 2010) and/or regulation of tight junction permeability along the axis of the gastrointestinal tract. However, for a more complete analysis of changes in paracellular permeability additional measurements with higher molecular markers (such as dextrans of different molecular weight) would be helpful.

A further explanation for the different effect of mast cell activation by ovalbumin contact between the colon and the small intestine might be differences in mast cell density. In contrast to the lung, where in our hands most of the staining techniques for mast cells worked quite well, staining of rat intestinal mast cells was only successfully by using an antibody against the surface marker c-kit (CD117), a receptor for stem cell factor (SCF). In principle, this receptor can be also found on the surface of intestinal Cajal cells, but in the intestine of rats these cells can be easily distinguished from mast cells because of their shape and location in or adjacent to the tunica muscularis. Indeed, when using this marker it turned out that mast cell density increased in jejunum from sensitized animals by approximately 230% in comparison to control animals, but tended to be reduced in the colon (Figure 4). A similar discrepancy between jejunum and colon was observed – as trend – when considering the expression of chymase (rMcpt-2) on the mRNA level. Thus, difference in mast cell density may be an explanation for the observation that only in the small intestine antigen exposure increased paracellular permeability. Additional induction of colitis did not alter mast cell density in either intestinal segment (Figure 4).

Sensitization against ovalbumin caused an increase in the protein level of claudin-3 and claudin-4 in immunofluorescent staining, which reached statistical significance in the morphometric analysis. Also the signal intensity of claudin-8 increased qualitatively, although we did not quantify this change due to the discontinuous immunofluorescent signal. All three proteins belong to the class of barrier-forming claudins (Günzel and Yu, 2013) and are stronger expressed in the colon compared to the small intestine as shown by Western blots (Markov et al., 2010). These results suggest a counterregulatory process in the OVA-sensitized animals, which – by enhancing the expression of barrier forming proteins – might serve to limit the entrance of antigens via the paracellular route. When in addition to the sensitization a colitis was induced, a significant decrease in claudin-3 immunofluorescence was observed in both intestinal segments, whereas the immunofluorescence for claudin-4 was unchanged in the colon, but increased significantly in the jejunum.

Taken together the present results demonstrate that at least in the TNBS/ethanol model of acute colitis in rats, there is no enhancement of antigen-induced ion secretion in the colon. In contrast, a slight downregulation of antigen-induced I_sc_ was observed, even in the small intestine suggesting systemic effects of circulating cytokines affecting immune response also in uninflamed areas of the gut. Whether changes in mast cell number and/or an altered mast cell mediator release or receptor composition are responsible for the altered anion secretion and how the response to antigen exposure might change in more chronic models of colitis are still to be investigated. An increase in the paracellular permeability measured as increase in fluorescein flux due to mast cell degranulation was only observed in the jejunum. This is a further example for the ability of mast cells to adapt to their microenvironment, e.g., by changing their mediator and receptor composition (Galli et al., 2008).

The reason for the unexpected missing upregulation of mast cell density in the sensitized animals with colitis remains speculative. However, in an *in vitro* cell culture model for mast cells incubated with proinflammatory cytokines we observed an increased apoptosis level measured as higher amount of caspase-3 positive cells compared to untreated cells (Becker and Diener, to be published). In contrast to the well-known relation that a reduced incidence of infections correlates with an increase in allergic diseases (Bach, 2002), we did not find any literature describing the reverse relation, i.e., possible changes of symptoms of food allergy during IBDs. So in future experiments it will be interesting to study in *in vivo* experiments whether allergic symptoms induced by oral challenge with ovalbumin in sensitized animals will be changed after previous induction of colitis or after specific infections, i.e., with intestinal parasites.

## Author Contributions

JB planned and performed the experiment and wrote the manuscript. DO performed the Western blots. MD planned the experiments and wrote the manuscript.

## Conflict of Interest Statement

The authors declare that the research was conducted in the absence of any commercial or financial relationships that could be construed as a potential conflict of interest.

## Footnotes

1 http://rsbweb.nih.gov/ij

2 http://www.hartfaalcentrum.nl
